# Genome-wide identification and analysis of ACP gene family in *Sorghum bicolor* (L.) Moench

**DOI:** 10.1186/s12864-022-08776-2

**Published:** 2022-07-25

**Authors:** Hanqiu Ge, Jingjing Xu, Mingzhu Hua, Wenwen An, Junping Wu, Baohua Wang, Ping Li, Hui Fang

**Affiliations:** 1 Ministry of Agricultural Scientific Observing and Experimental Station of Maize in Plain Area of Southern Region, School of Life Sciences, Nantong University, Nantong, 226019 Jiangsu People’s Republic of China; 2 Nantong Changjiang Seed Co., Ltd, Nantong, 226368 Jiangsu People’s Republic of China

**Keywords:** *Sorghum bicolor* (L.) Moench, Gene family analysis, ACP genes, Bioinformatics

## Abstract

**Background:**

Acyl carrier proteins (ACP) constitute a very conserved carrier protein family. Previous studies have found that ACP not only takes part in the fatty acid synthesis process of almost all organisms, but also participates in the regulation of plant growth, development, and metabolism, and makes plants adaptable to stresses. However, this gene family has not been systematically studied in sorghum.

**Results:**

Nine ACP family members were identified in the sorghum genome, which were located on chromosomes 1, 2, 5, 7, 8 and 9, respectively. Evolutionary analysis among different species divided the ACP family into four subfamilies, showing that the SbACPs were more closely related to maize. The prediction results of subcellular localization showed that SbACPs were mainly distributed in chloroplasts and mitochondria, while fluorescence localization showed that SbACPs were mainly localized in chloroplasts in tobacco leaf. The analysis of gene structure revealed a relatively simple genetic structure, that there were 1–3 introns in the sorghum ACP family, and the gene structure within the same subfamily had high similarity. The amplification method of SbACPs was mainly large fragment replication, and SbACPs were more closely related to ACPs in maize and rice. In addition, three-dimensional structure analysis showed that all ACP genes in sorghum contained four α helices, and the second helix structure was more conserved, implying a key role in function. *Cis*-acting element analysis indicated that the *SbACPs* might be involved in light response, plant growth and development regulation, biotic and abiotic stress response, plant hormone regulation, and other physiological processes. What’s more, qRT-PCR analysis uncovered that some of SbACPs might be involved in the adaptive regulation of drought and salt stresses, indicating the close relationship between fatty acids and the resistance to abiotic stresses in sorghum.

**Conclusions:**

In summary, these results showed a comprehensive overview of the SbACP*s* and provided a theoretical basis for further studies on the biological functions of SbACPs in sorghum growth, development and abiotic stress responses.

**Supplementary Information:**

The online version contains supplementary material available at 10.1186/s12864-022-08776-2.

## Background

Acyl Carrier Protein (ACP) is a class of acidic proteins with low molecular weight and conserved serine residues [1, 2]; the serine residues on ACP proteins are covalently linked to phosphopantetheine linker, which is connected to the lipoyl group through the -SH group [3, 4]. The site-directed mutation was used to mutate the binding site (Ser38) of spinach acyl carrier protein I (ACP-I) from serine to threonine or cysteine residues, which changed the conformation of ACP and led to the loss of ACP function [1], suggesting that this locus had an important biological function. ACP is an important cofactor of fatty acid synthase. There are two different types of type II fatty acid synthases in plant cells. One is the cytoplasmic fatty acid synthase, which is responsible for most fatty acid synthesis, and the other is located in the mitochondria, which produces the fatty acid precursors necessary for the production of lipoic acid. ACPs-loaded lipoyl groups shuttle back and forth across various functional sites of fatty acid synthase to perform functions [5–8].

Functionally, ACPs are involved in the metabolism of different types of fatty acids in plants, such as biosynthesis of fatty acids, polyketones and non-ribosomal proteins [9, 10], and also involved in responses to biological and abiotic stresses [11–15]. The unsaturated fatty acids (UFAs) of membrane lipid also adjust to environmental conditions by changing the fluidity of membrane lipids. Besides the production of saturated fatty acids (SFAs), ACPs are also involved in the biosynthesis of UFAs. The Arabidopsis fatty acid desaturase2, an endoplasmic reticulum (ER)-localized ω-6 desaturase for converting oleic acid to linoleic acid, was necessary for chilling and salt tolerance [16, 17]. Compared with mature leaves, the mRNA level of ACP in young leaves of spinach and soybean was significantly higher, while compared with leaves grown under dark conditions, spinach leaves grown under light also contained higher ACP activity, suggesting that ACP might be related to plant growth and development and light response [18]. *Brassica napus* could encode multiple copies of ACPs to meet the needs of fatty acid synthesis that occurred during oilseed development [19]. *AhACP1* was mostly expressed in peanut seeds, and *AhACP4* and *AhACP5* showed the same mRNA expression profile in different organs and seeds during development. Two highly expressed mitochondrial ACPs were highly expressed in peanut flower tissues [20]. Besides, combining linkage analysis, whole-genome analysis, candidate gene association analysis and plant transformation, *GmACP1*, a candidate gene encoding acid phosphatase in hairy roots of soybean, was identified to improve soybean tolerance to low phosphorus stress [21]. In *Arabidopsis thaliana*, the function of ACP genes had also been extensively studied. AtACP genes could respond to several abiotic stresses, e.g., the expression of *AtACP1*, *AtACP2* and *AtACP3* could be induced by drought. The expression of *AtACP4* was down-regulated by both iron and nitrogen deficiency. The expression of *AtACP5* decreased significantly after salt stress. Overexpression of *AtACP5* further led to changes in the composition of fatty acids, mainly including the decrease of oleic acid (C18:1) and increase of palmitic acid (C16:0); in addition, the ratio of sodium to potassium was also significantly lower than that of the wild type [14]. Recently, it was found that wheat chloroplast acyl carrier protein synthase I and chloroplast 20 kDa chaperone proteins significant increased under water stress [15]. All these studies demonstrated the diversity of ACP gene functions.

At present, agricultural production and food security in developing countries are still facing many threats. Sorghum [*Sorghum bicolor* (L.) Moench], as the fifth-largest food crop in the world and C4 crops, with high photosynthetic efficiency and a developed root system, is an important crop variety in arid and semi-arid regions with wide uses [22–25]. The importance of drought-tolerant crops like sorghum may increase as some areas unsuitable for rice and maize cultivation due to rising temperatures and decreasing precipitation caused by climate change [26]. Currently, sorghum breeding focuses on improving adaptation to climate change, mainly including biotic and abiotic stresses [27–32]. With the development of high-throughput sequencing, whole genomes of many species have been released. Paterson et al. completed the assembly of the whole sorghum genome and the data are available in the United States Department of Energy (DOE) Joint Genome Institute (JGI) [33]. Meanwhile, genome assembly has been completed in multiple plants including Arabidopsis, soybean, peanut, olive rape, etc., which greatly facilitates the analysis of gene family, and ACP gene families have been characterized in multiple species [19, 20, 34–36]. However, genome-wide analysis of the ACP gene family in sorghum has not been reported. In this study, the ACP gene family was systematically identified and analyzed at the genome level, and the expression patterns of ACPs under two kinds of abiotic stresses including drought and salt stress were also analyzed, which laid a foundation for the further investigation of the molecular mechanism of ACP in response to stress.

## Results

### Identification and sequence analysis of ACP gene family in sorghum

**Fig. 1:**
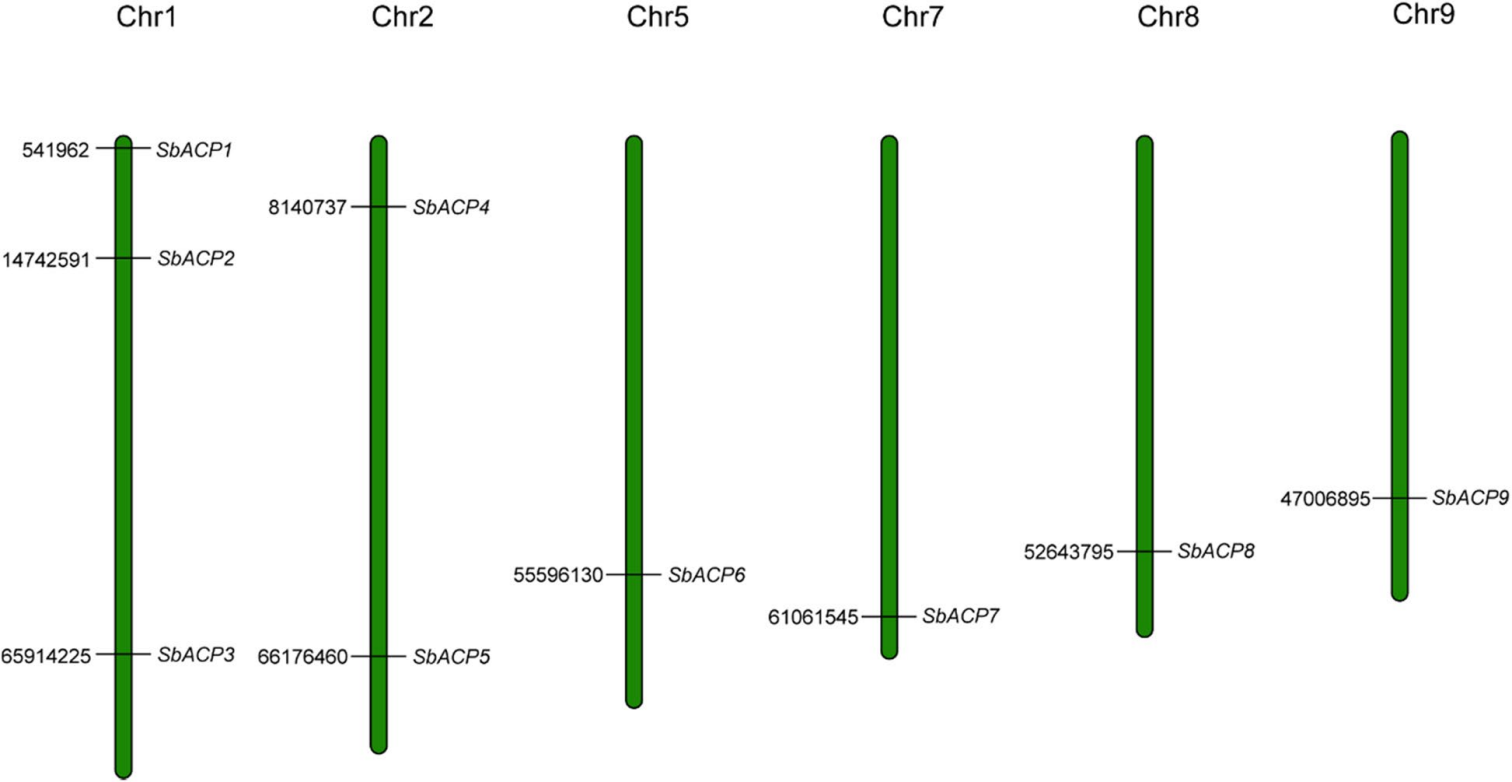
Chromosomal location of ACP genes in sorghum. The number on the left of chromosome is physical position of genes

Nine ACP gene family members were screened and identified from sorghum genome database and named as *SbACP1*-*SbACP9* [37]. The molecular weights of the nine ACP genes ranged from 14,443.48 to 15,727.81 kDa, and the theoretical isoelectric point ranged from 4.68 to 5.67. All nine ACP genes were acidic (PI < 7), so the ACP family in sorghum was a kind of acidic proteins with low molecular weight. Nine ACP family genes were distributed on chromosomes 1, 2, 5, 7, 8 and 9. Of which, *SbACP1*, *SbACP2* and *SbACP3* were located on chromosome 1, and *SbACP4* and *SbACP5* were located on chromosome 2, while *SbACP6*, *SbACP7*, *SbACP8*, and *SbACP9* were distributed on chromosome 5, 7, 8, and 9, respectively (Fig. 1). In addition, the detailed information of these genes was shown in Table 1.

**Table 1:** Protein sequence information of Sorghum ACP family genes in almonds

Name	gene_ID	mRNA_ID	Chromosome location	Length (AA)	Mw (kD)	PI
SbACP1	SORBI_3001G005700	EER90483	Chr1:541,962..544103 +	146	15,727.81	4.68
SbACP2	SORBI_3001G175600	EER91268	Chr1:14,742,591..14747705 +	142	15,346.3	5.21
SbACP3	SORBI_3001G370700	EER92267	Chr1:65,914,223..65918203 +	131	14,443.48	5.37
SbACP4	SORBI_3002G078300	EER96074	Chr2:8,140,737..8141673 +	134	14,526.69	5.41
SbACP5	SORBI_3002G280400	EER99269	Chr2:66,176,461..66179180-	135	14,266.39	5.06
SbACP6	SORBI_3005G128100	EES08540	Chr5:55,596,128..55599665 +	143	15,127.31	5.19
SbACP7	SORBI_3007G176800	EES14114	Chr7:61,061,543..61064169 +	131	13,843.82	4.84
SbACP8	SORBI_3008G116400	EES17130	Chr8:52,643,796..52646872-	142	15,143.26	5.67
SbACP9	SORBI_3009G119900	EES19437	Chr9:47,006,894..47010121-	128	13,829.76	5.54

### Subcellular localization of SbACPs

**Fig. 2:**
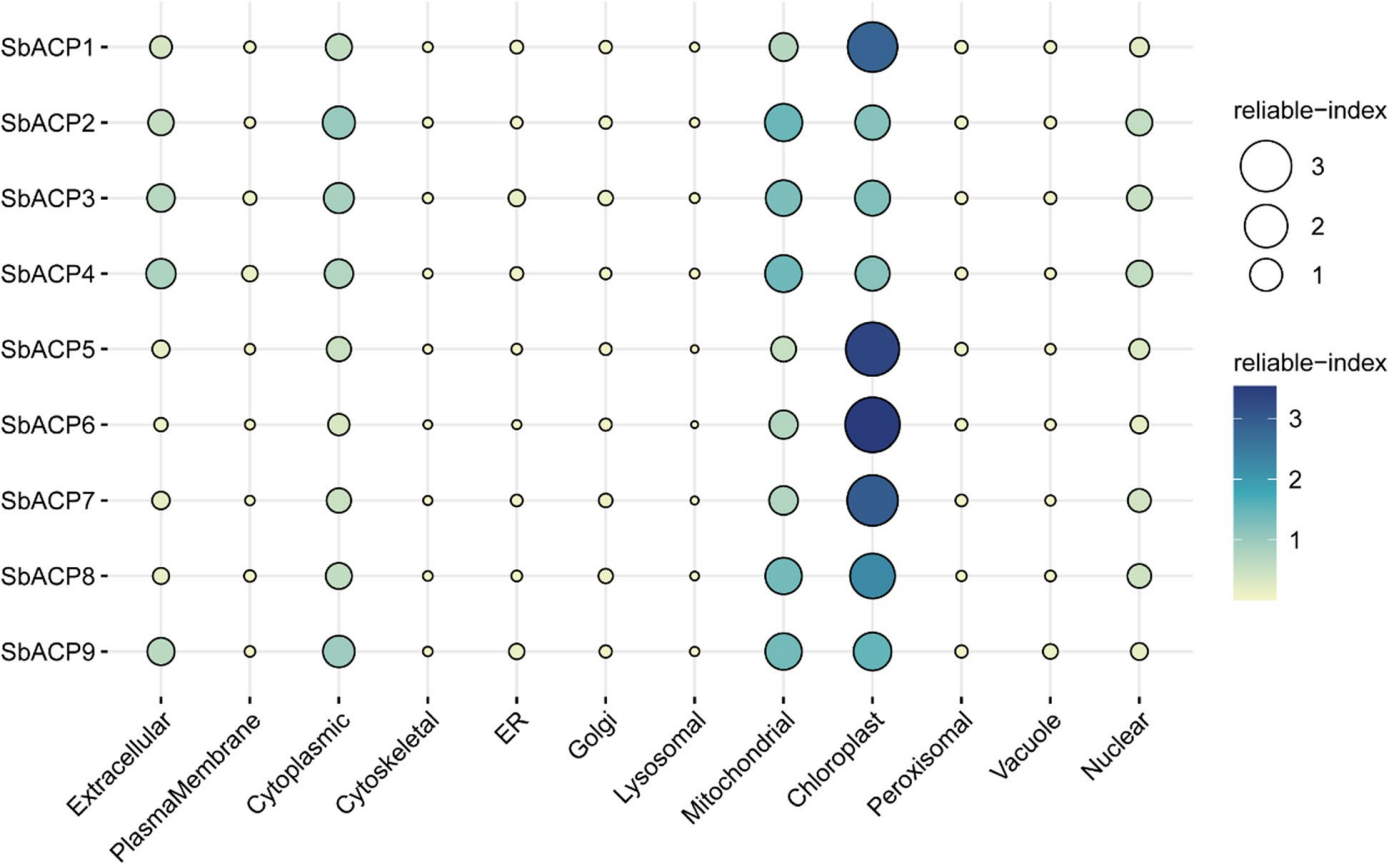
The prediction of subcellular localization for ACP genes in sorghum. The color and the size of the circle indicate the values of reliable index of the prediction results

**Fig. 3:**
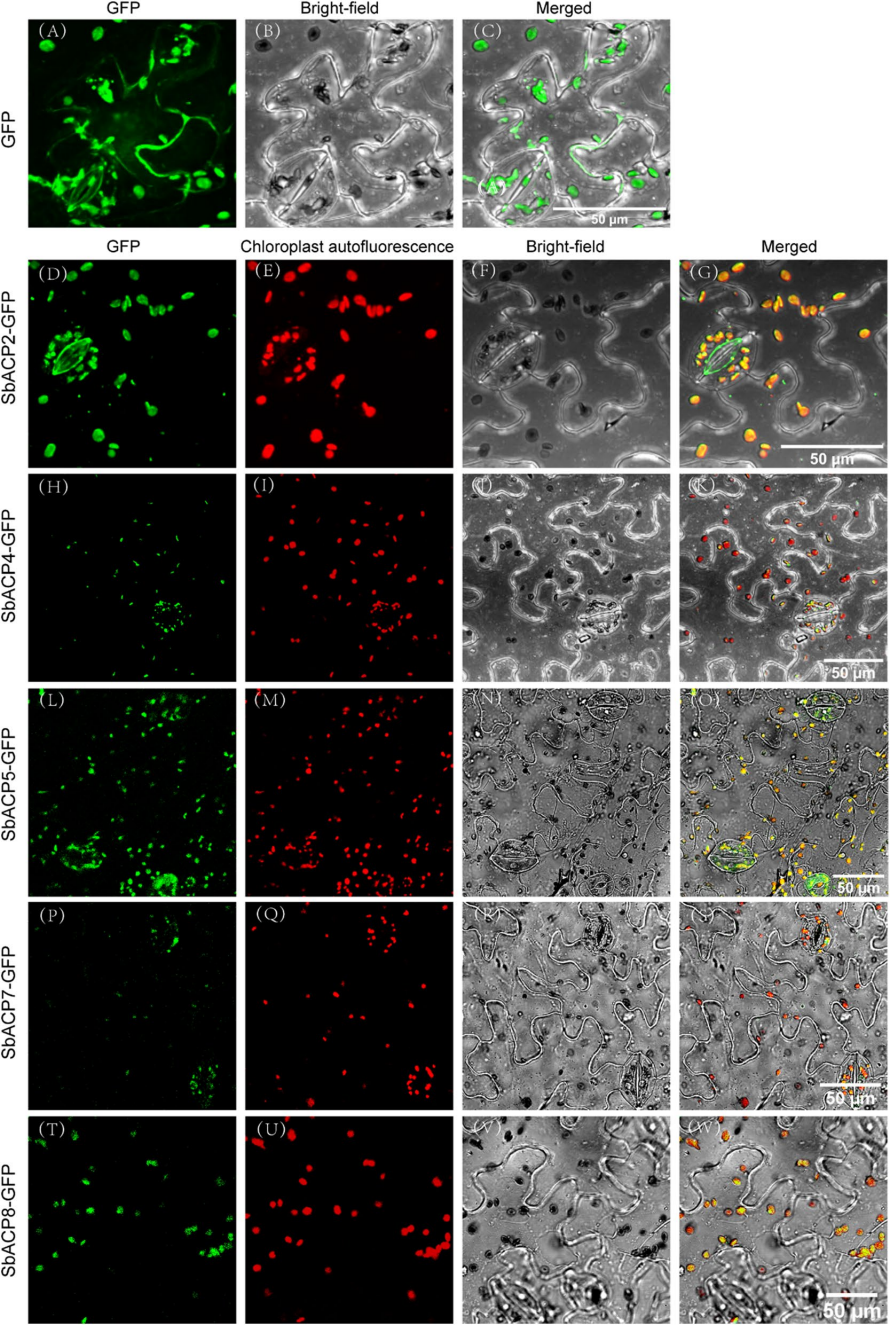
Subcellular localizations of SbACPs in tobacco leaf epidermal cells by confocal laser-scanning microscopy. The 5 selected SbACP-GFP fusion proteins were predominantly localized to the chloroplast. GFP, green fluorescent protein

To figure out where the ACP proteins were expressed, the prediction of subcellular localization was carried out and the results showed that SbACP2, SbACP3, SbACP4 and SbACP9 were highly likely to be expressed in mitochondria or chloroplasts, whose values of reliable index were very close, while the other SbACPs were highly possible to be located in chloroplasts, with the values of reliable index expressed in chloroplasts significantly higher than that in other tissues, indicating that the ACP proteins probably functioned in mitochondria or chloroplasts. (Fig. 2). To verify the reliability of the prediction, five SbACPs with GFP tags were selected to determine their expression localization in tobacco leaf epidermal cells. Confocal fluorescence results showed that all the 5 SbACPs were located in chloroplasts (Fig. 3).

### Evolution analysis of SbACP Proteins

**Fig. 4:**
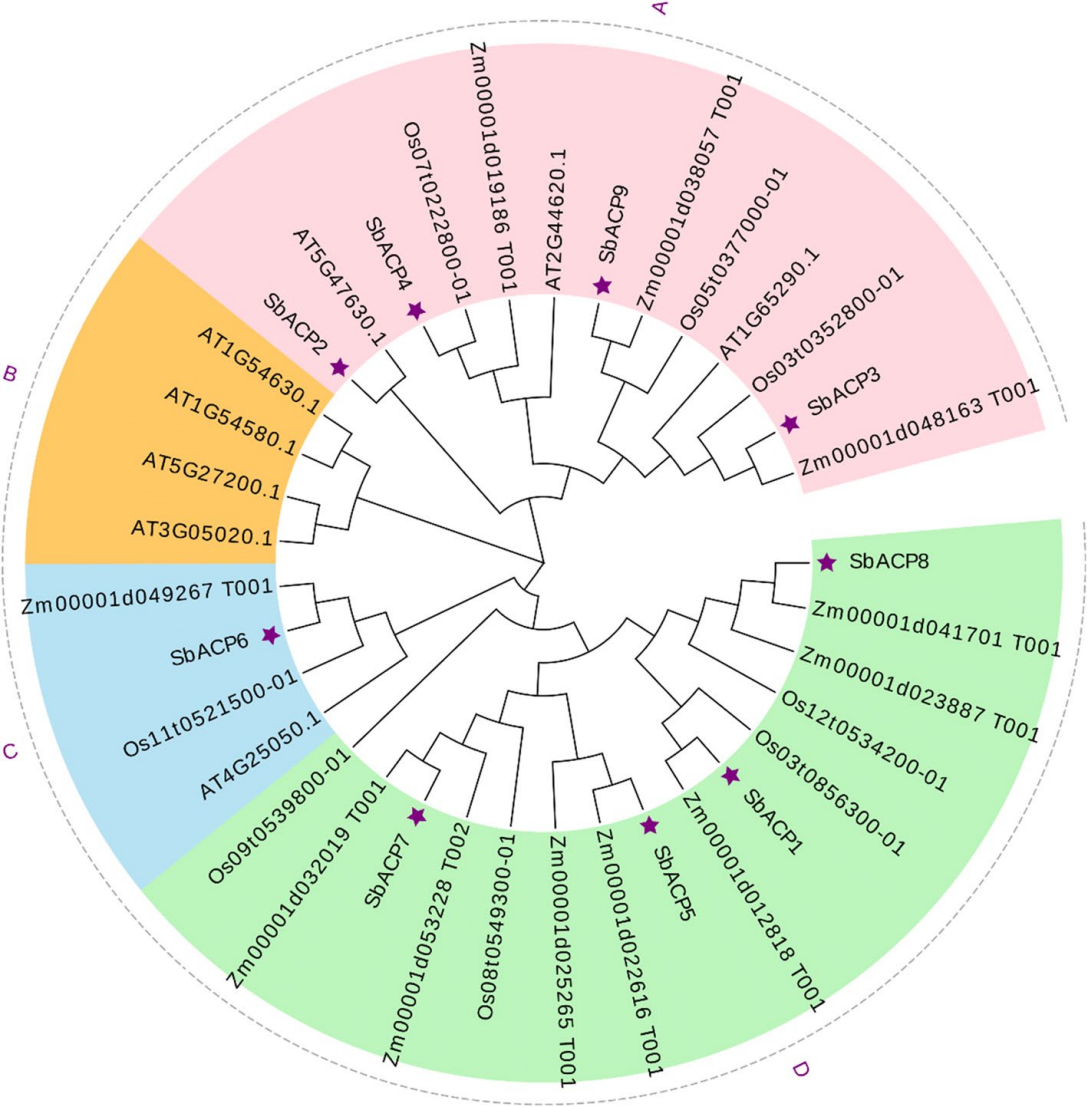
Clustering of SbACPs in three species. Sb: sorghum; At: Arabidopsis; Zm: maize. All protein members were divided into four clusters: cluster A on pink background, cluster B on blue background, cluster C on orange background and cluster D on green background. The genes marked with purple asterisk were the members of sorghum ACP family

In order to clarify the evolutionary relationship and obtain more detailed classification of ACP proteins in sorghum, maize, rice and Arabidopsis, the phylogenetic tree including 36 ACP proteins were constructed. Based on the alignment of full-length protein sequences of 9 ACPs in sorghum, 11 ACPs in maize, 8 ACPs in rice and 8 ACPs in Arabidopsis, these proteins were categorized into 4 different subfamilies named as A, B, C and D (Fig. 4). Cluster A and D contained 13 and 15 ACPs, respectively, while cluster B and C covered 4 ACPs, respectively. Further, SbACPs were distributed in clusters A, C and D, with SbACP1, SbACP5, SbACP7 and SbACP8 in cluster A, SbACP2, SbACP3, SbACP4 and SbACP9 in cluster D, and only SbACP6 in cluster C. Only 4 ACPs of Arabidopsis were contained in cluster B, suggesting a relative distant evolutionary relationship of ACPs between Arabidopsis and other three species, implying the difference of ACP gene sequences between monocotyledon and dicotyledon. Furthermore, ACP proteins in sorghum and maize were always in close branches in clusters A, B and D, implying a closer evolutionary relationship of ACPs in sorghum and maize that are both C4 plants, and followed by sorghum and rice.

### Gene structure and conserved motif analysis of SbACP proteins

**Fig. 5:**
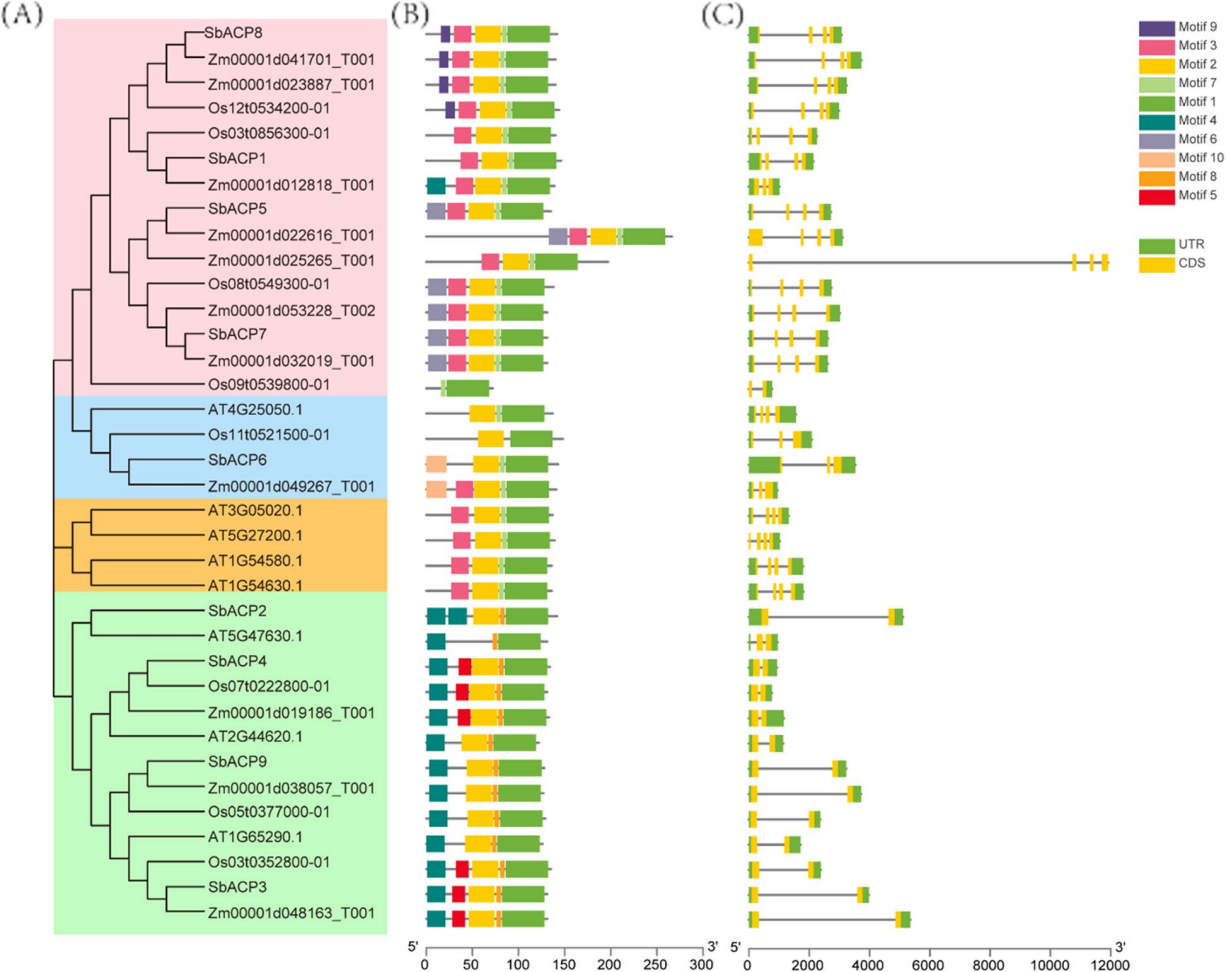
Phylogenetic tree (**A**), conserved motifs (**B**) and gene structure (**C**) analysis of SbACP family. The scaleplates under **B** and **C** indicate the length of SbACP proteins and genes, respectively

In order to study the exon–intron organization of individual SbACP gene, the GSDS website was used to predict the gene structure of SbACPs. Based on the phylogenetic tree, the SbACP genes were clearly classified into three major subfamilies (cluster A, C and D). The analysis of gene structure suggested that the members within the same family possessed the similar exon–intron structure (Fig. 5C). Of all 9 SbACP genes, 4 genes belonging to cluster A, including *SbACP2*, *SbACP3*, *SbACP4* and *SbACP9* were relatively simple in structure, each had 2 exons and 1 intron. For another 4 genes in cluster D, namely *SbACP1*, *SbACP5*, *SbACP7* and *SbACP8*, each of them contained 4 exons and 3 introns. The remaining *SbACP6* contained three exons and two introns, which belonged to cluster C. These results further verified the reliability of phylogenetic tree and indicated that genes in the same clusters tended to be more similar in genetic structure including the number of exon and intron, but might be with different intron length. Meanwhile, the diversity of gene structure among SbACP genes indicated that different selection events might have taken place during gene evolution.

The conserved motifs were often closely related to protein function involved in protein–protein interactions, nuclear localization, and transcriptional activities [38]. Therefore, to uncover the characteristic regions of ACP proteins, MEME software was used to analyze the conserved motif of ACP proteins. Totally 10 motifs were identified and designated as Motif 1–10 (Fig. 5B). Among them, Motif 1 and Motif 2 existed in all ACP family members, Motif 3 and Motif 7 existed in cluster B, cluster C and cluster D; Motif 4, Motif 5 and Motif 8 existed in cluster A; Motif 9 and Motif 10 existed only in cluster A and cluster B, respectively (Fig. 5B). Combining the analysis of conserved motifs, evolutionary tree, and gene structure, we found that genes within the same subfamily tended to share similar gene structure and motif, and meanwhile, we inferred that the proteins within the same cluster containing the similar composition of conserved motifs might share the similar function. Taken together, all these results further confirmed the classification of SbACP proteins in sorghum.

### Duplication and syntenic analyses in sorghum ACP genes

**Fig. 6:**
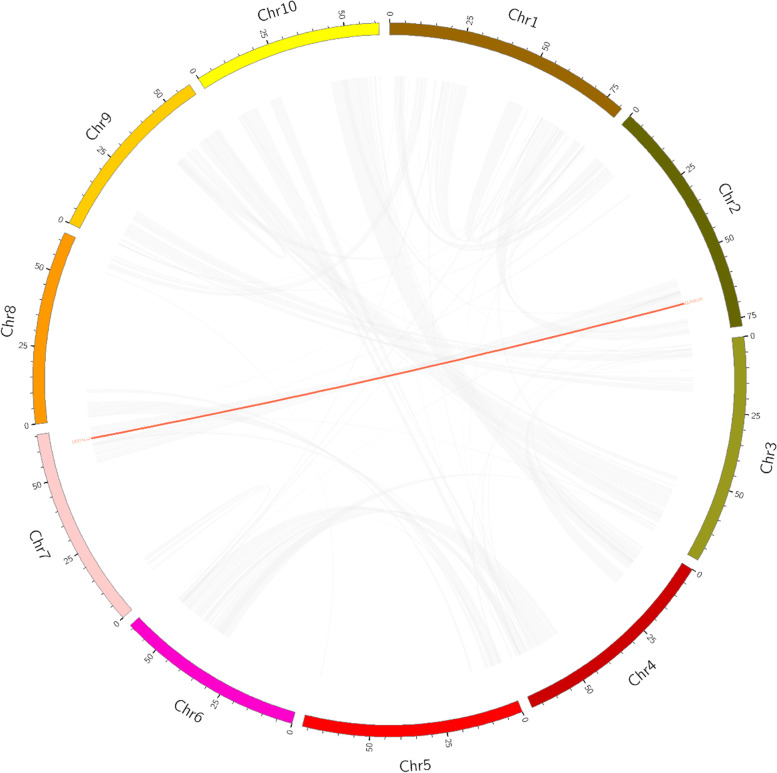
Interchromosomal relationships of SbACPs genes. Grey lines indicate all syntenic blocks in the sorghum genome. Red lines indicate collinear blocks of ACP genes in the sorghum genome

Genome replication events have long been considered the origin of evolutionary novelty. During evolution, gene duplication, tandem duplication and large fragment duplication tend to initiate the generation of gene families. Therefore, we analyzed the gene duplication events of the SbACP genes in sorghum. All nine SbACPs were so far apart from each other on the genome that no tandem repetition occurred (Fig. 1). However, we found that a pair of ACP genes were segmented repeats (SbACP7/SbACP5) (Fig. 6). These results indicated that most of the SbACP genes might evolve independently, and segment repeats play a slightly role in contributing to the expansion of the SbACP gene family.

**Fig. 7:**
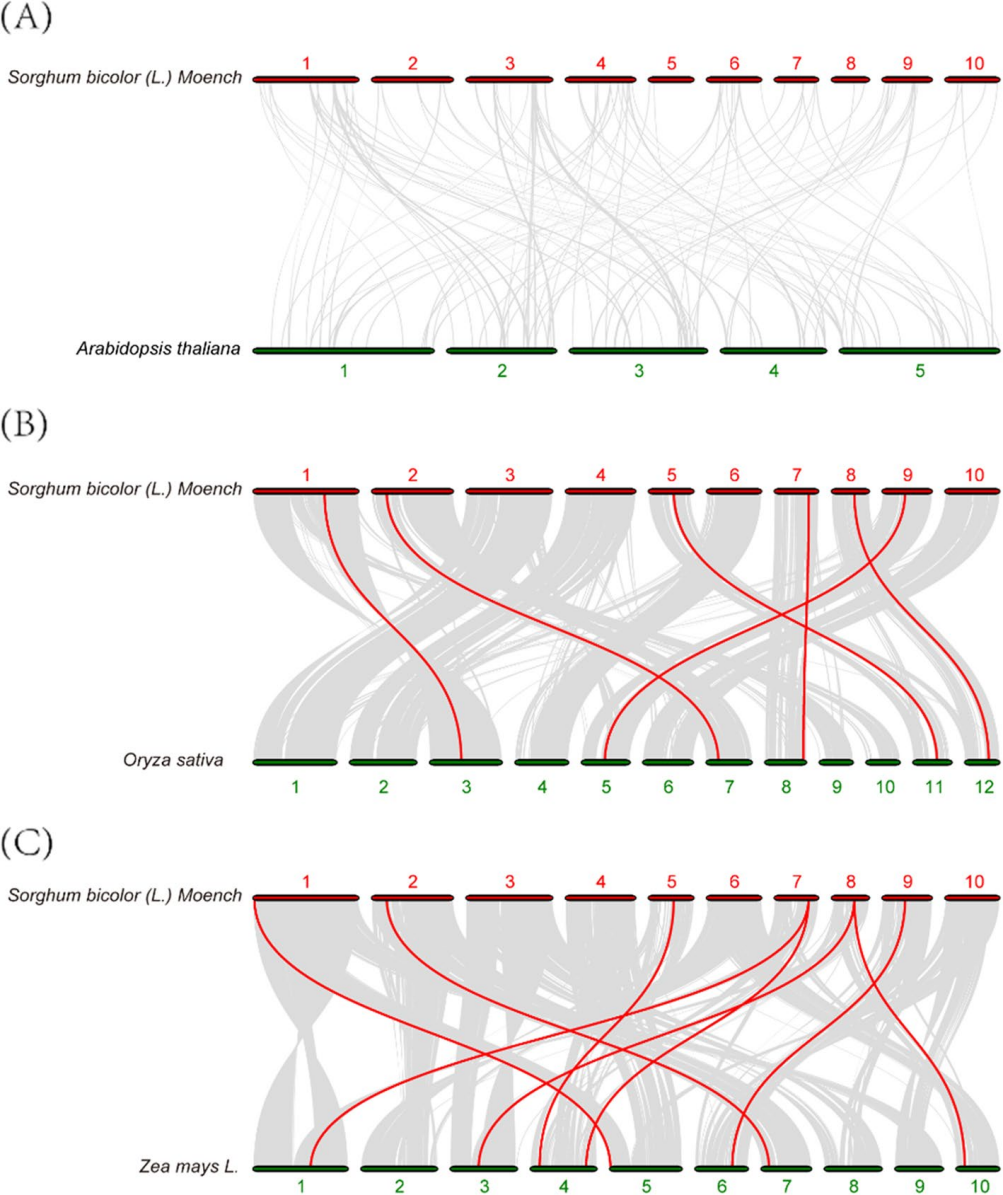
Syntenic analysis of ACP genes between sorghum and three representative plant species. **A** Syntenic analysis of ACP genes between sorghum and Arabidopsis. **B** Syntenic analysis of ACP genes between sorghum and rice. **C** Syntenic analysis of ACP genes between sorghum and maize. Gray lines in the background indicate the collinear blocks within sorghum and other plant genomes, whereas red lines highlight syntenic ACP gene pairs

In order to further explore the evolutionary information of ACP genes between sorghum and other species, collinearity analysis was respectively performed between the genome of sorghum and three other plants, including one dicotyledons (Arabidopsis) and two monocotyledons (rice and maize). From the results, we found 0, 6, and 8 collinear gene pairs in sorghum with Arabidopsis, rice, and maize, respectively (Fig. 7). Five SbACPs (*SbACP4*, *SbACP6*, *SbACP7*, *SbACP8*, *SbACP9*) have collinear pairs in all two monocot species and no collinearity of ACPs existed between sorghum and Arabidopsis, indicating evolution divergence of ACP gene family between monocotyledons and dicotyledons. Simultaneously, we speculated that SbACPs might have evolved from homologous genes of other monocotyledons.

### Multiple sequence alignment and three-dimensional structure prediction of SbACP proteins

**Fig. 8:**
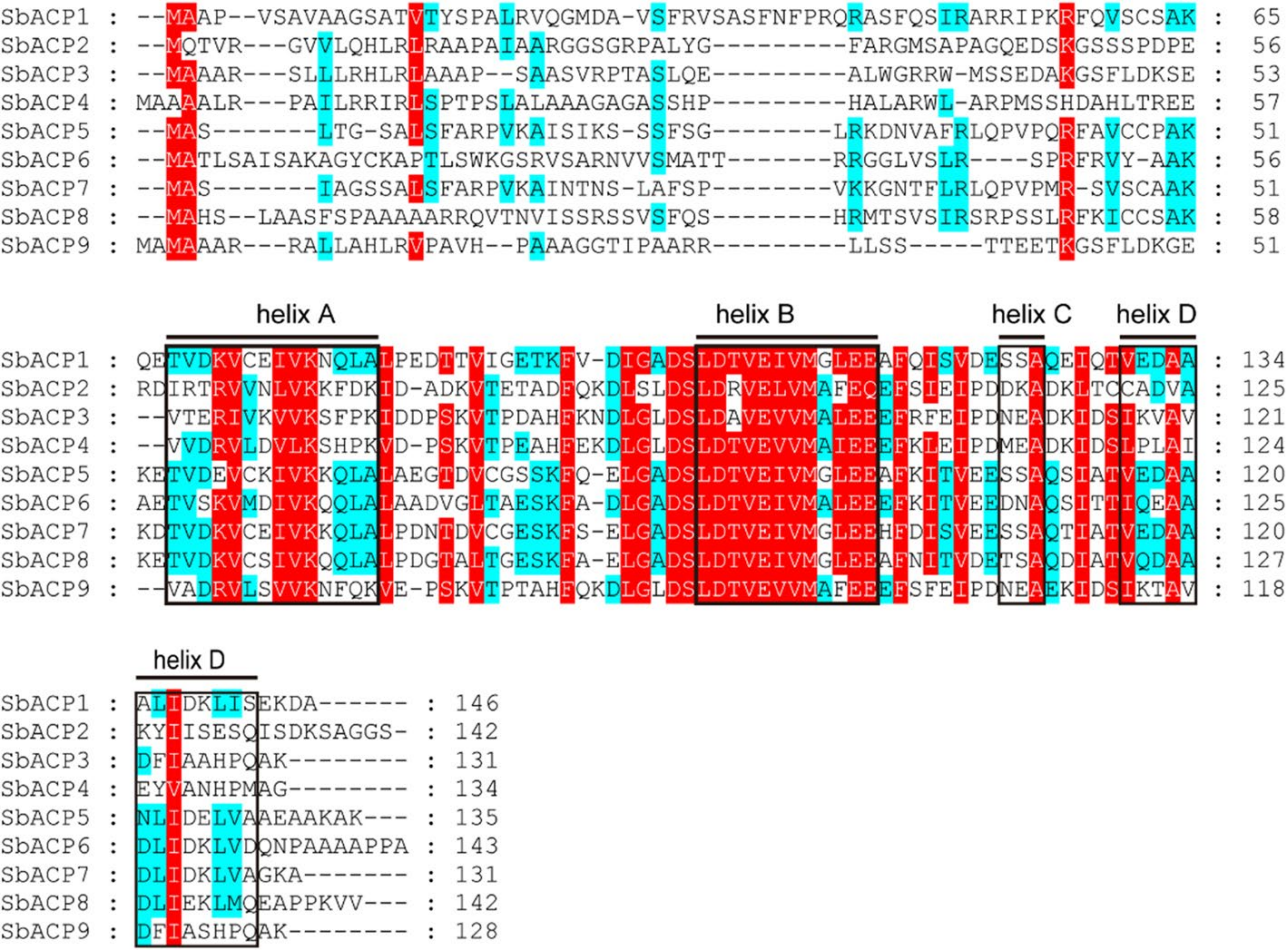
Conserved domains of SbACP gene family. The red background stands for conservative amino acids, and the skyblue background stands for less conservative amino acids. The black boxes represent alpha spirals (helix A, helix B, helix C, helix D)

In order to further identify the sequence feature of ACP conserved domain, the protein sequences of 9 SbACP genes were aligned and analyzed. The results of alignment suggested that 4 domains of SbACP including Helix A-D were relatively conserved in sorghum (Fig. 8), especially the sequences around the Asp-Ser-Leu (DSL) motif (Helix B), which was the binding site of phosphopantetheine. This indicated that the DSL motif might have an important biological function.

**Fig. 9:**
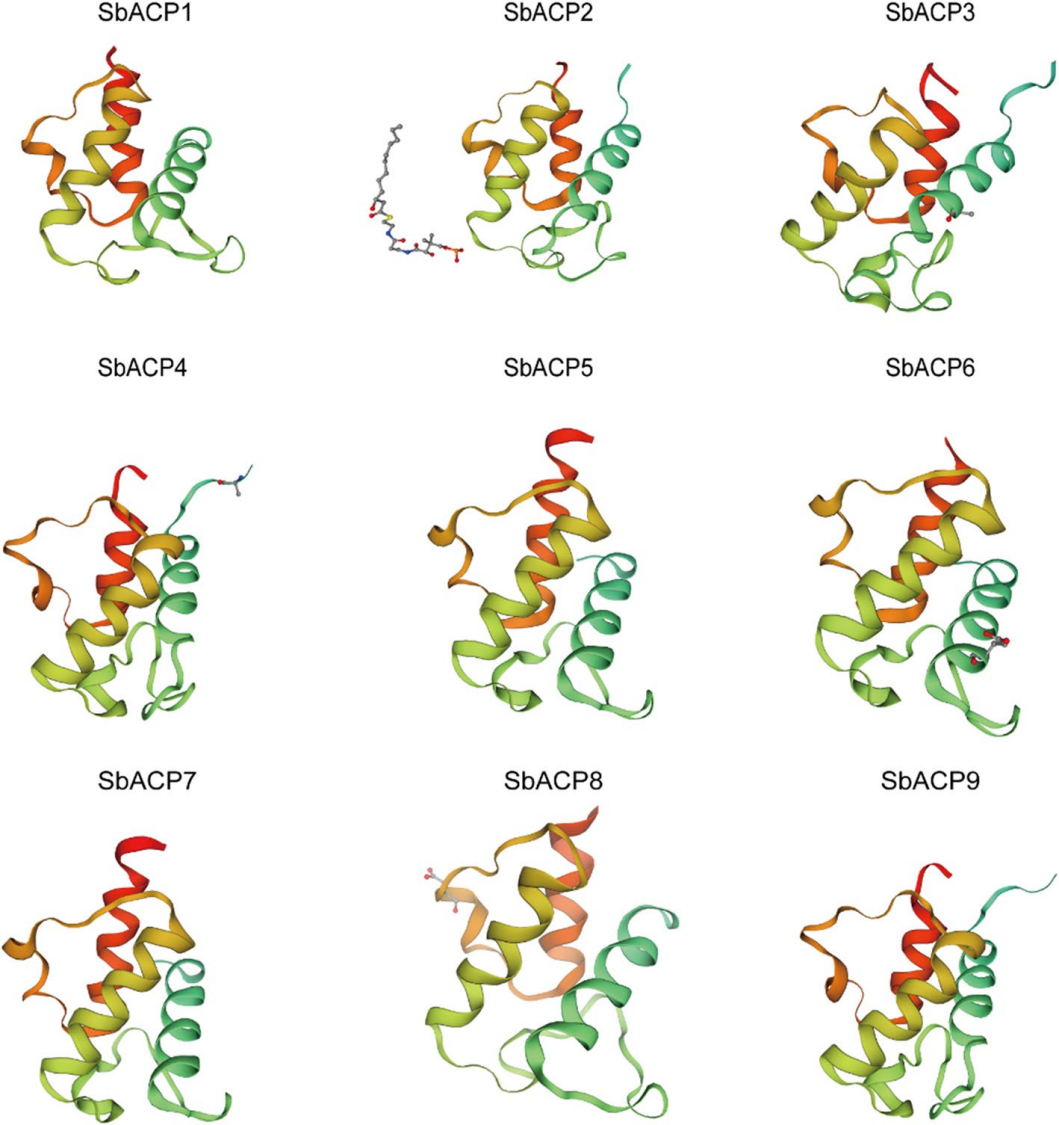
Prediction of three-dimensional structure of ACP proteins in sorghum

Protein conformation was often related to their function. In order to further understand the function of the SbACP proteins, their three-dimensional structures were predicted through SWISS-MODEL website (Fig. 9). The results showed that more than 50% of the secondary structure of SbACP proteins was α-helix. SbACPs contained 4 α-helix structures, with A helix parallel to B helix and D helix, and C helix connecting B helix and D helix was relatively short. The results of multiple sequence alignments showed that B helix was the most conserved, while A, C and D helix were relatively less conserved. The binding site of phosphopantetheine was found at the nitrogen end of B helix, which might be the reason why the second helix was so conserved.

### The *cis*-acting element of SbACPs

**Fig. 10:**
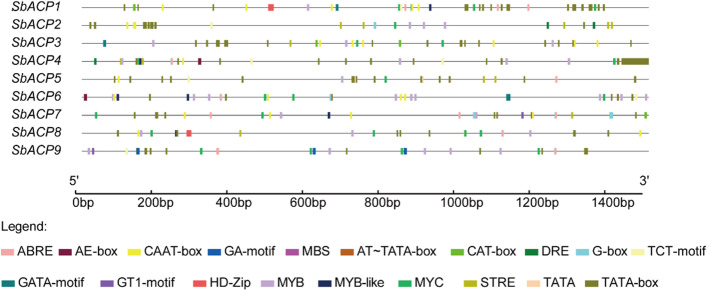
Analysis of *cis*-acting elements in 1.5 kb promoter regions of SbACP genes

In order to explore the potential molecular function of SbACP gene family, the 1.5 kb promoter sequences upstream of SbACP genes were analyzed to detect the *cis*-acting elements on Plant CARE website. The results suggested that a variety of cis-acting elements involved in physiological processes were revealed, including some basic elements like TATA-box and CAAT-box, (Fig. 10). For example, the *cis*-acting elements AE-box, GA-Motif, G-box, TCT-motif, GATA-motif, and GT1-motif were involved in light reaction; the binding sites of *cis*-acting elements ABRE and MYB were involved in abscisic acid response; MBS was involved in drought induction, and *cis*-regulatory element CAT-box was associated with meristem expression. These results clearly suggested that SbACP genes might participate in regulating biotic or abiotic stress, light response, plant growth and development, and signaling transduction pathways, as well as other physiological processes.

### Expression patterns of SbACPs gene family under drought and salt stress

**Fig. 11:**
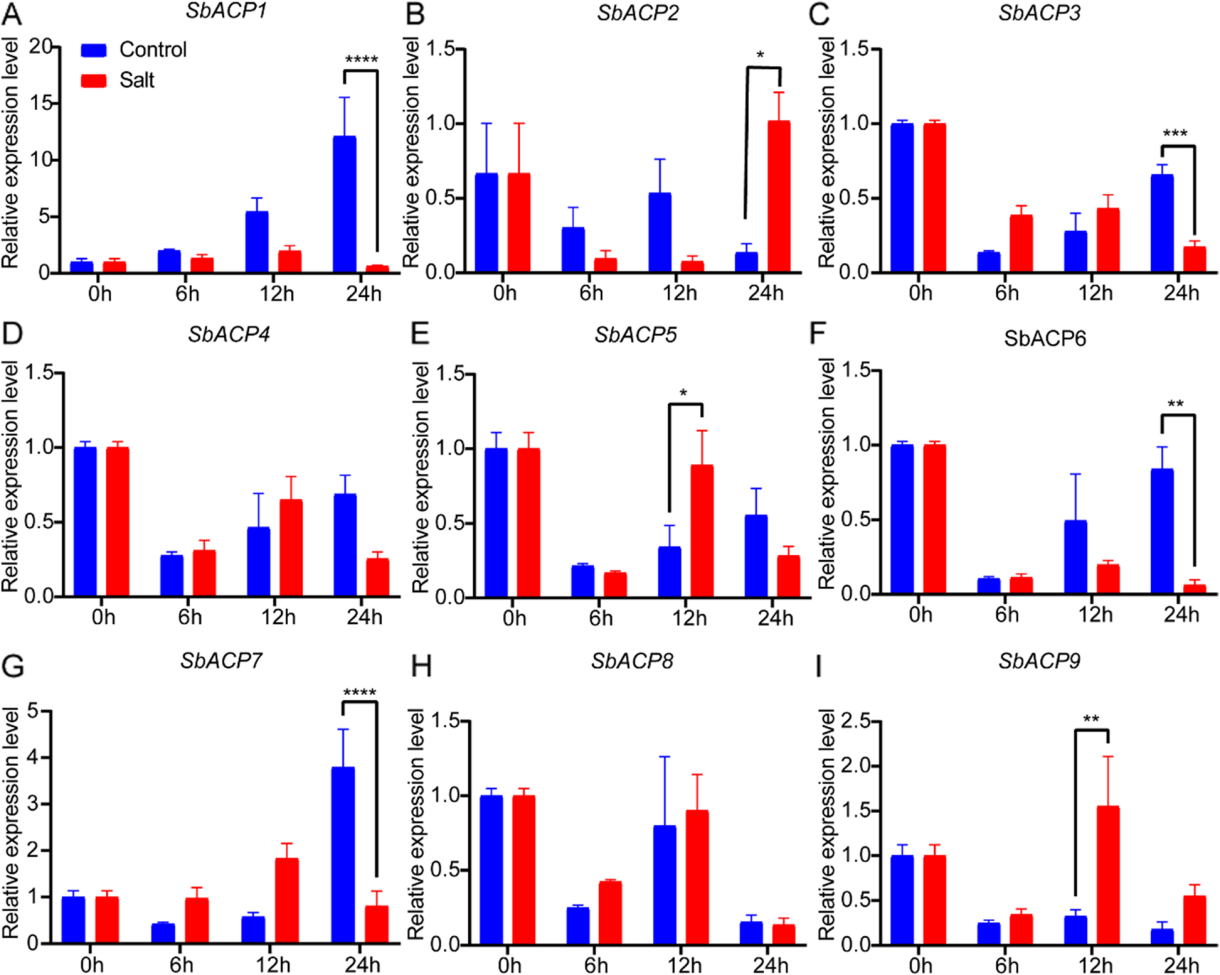
qRT-PCR analysis of SbACPs under salt treatment. *, **, *** and ****, represent significant differences at *P* < 0.05, *P* < 0.01, *P* < 0.001 and *P* < 0.0001, respectively. One-way ANOVA was used to analyze the significance of relative expression difference of SbACP1-9 at 0, 6, 12 and 24 h under salt treatment and control

**Fig. 12:**
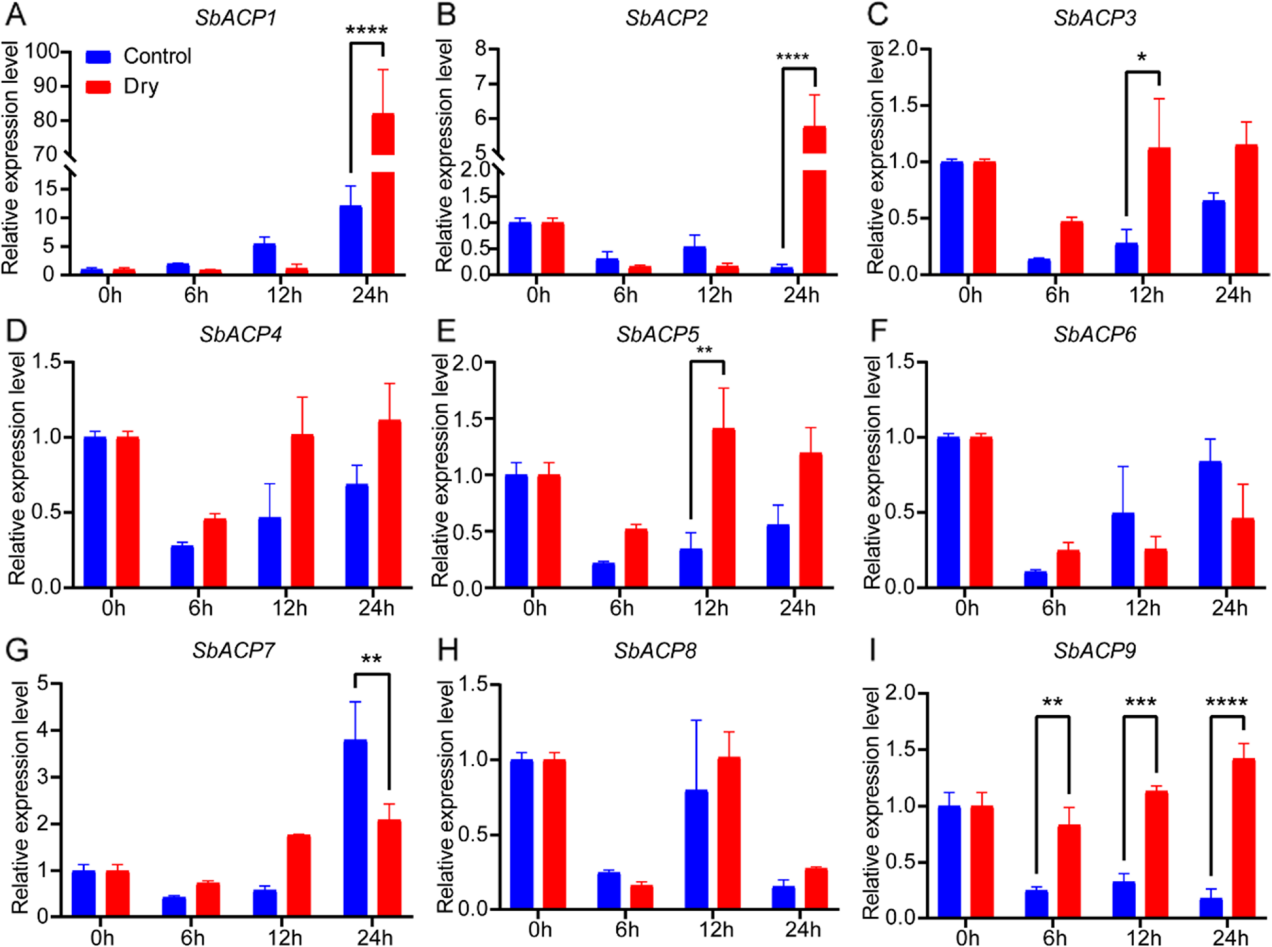
qRT-PCR analysis of SbACPs under drought treatment. *, **, *** and ****, represent significant differences at *P* < 0.05, *P* < 0.01, *P* < 0.001 and *P* < 0.0001, respectively. One-way ANOVA was used to analyze the significance of relative expression difference of SbACP1-9 at 0, 6, 12 and 24 h under drought treatment and control

Previous studies have proved that ACP genes played critical roles in response to abiotic stresses in Arabidopsis [14], and the identification of *cis*-acting elements related to abiotic stress response in SbACPs promoter regions also indicated their potential function involved in different abiotic stress response pathways. Meanwhile, gene expression patterns could provide important clues to gene function. Consequently, to further confirm molecular function of SbACP genes in response to abiotic stress, the expression levels of SbACP genes in sorghum leaves were analyzed by qRT-PCR at 0, 6, 12 and 24 h after drought and salt treatment (Figs. 11 and 12). The results showed that seven of the nine SbACP genes were up-regulated or down-regulated within 24 h of drought and salt treatment, implying that these genes might play a role in response to drought and salt stress. Still, no significant expression differences of *SbACP4* and *SbACP8* were observed when compared with the control under drought and salt stress, indicating that these two genes were not directly involved in stress responses. Furthermore, we found that different genes showed different expression patterns under stresses. Under salt stress, *SbACP1*, *SbACP3*, *SbACP6* and *SbACP7* were significantly down-regulated at 24 h after treatment, whereas *SbACP2* was significantly up-regulated. *SbACP5* and *SbACP9* were up-regulated at 12 h. Under drought stress, *SbACP3*, *SbACP5* and *SbACP9* were significantly up-regulated at 12 h, *SbACP1*, *SbACP2* and *SbACP9* were significantly up-regulated at 24 h, whereas *SbACP7* was significantly down-regulated at 24 h. It's worth noting that, of all genes with expression difference, *SbACP1* showed the greatest fold of expression difference at 24 h after salt treatment, while under drought treatment, the expression of *SbACP1* at 24 h was up-regulated up to five times. Interestingly, *SbACP9* was continuously up-regulated after 6 h of drought treatment. In a word, these results revealed that most of SbACP genes could take part in response to the two abiotic stresses, but might be with different mechanisms due to their different expression patterns.

## Discussion

With the development of sequencing technology, large numbers of plant genomes have been successfully assembled and released, which has greatly improved the research of genomes, including analysis of gene families and identification of important functional genes. Acyl carrier proteins are important members of the carrier protein family and play a key role in the direct synthesis pathway of long-chain fatty acids [13, 39].

Gene family is a group of genes that originate from the same ancestor and produce two or more copies of one gene through gene duplication [40]. They have obvious similarities in structure and function and encode similar protein products. There were 9 ACPs in sorghum, 8 ACPs in rice, 11 ACPs in maize and 8 ACPs in Arabidopsis. During gene evolution, gene duplication or loss may lead to differences in the number of gene family members [41]. In this study, nine SbACPs were identified from sorghum genome by bioinformatics methods, and their physicochemical properties were predicted to be a class of acidic small molecule proteins, which was consistent with previous reports [42]. The protein length, molecular weight, and isoelectric point of SbACPs were highly conservative, whereas the number of exons varied from 2 to 4. Multi-sequence alignment of the nine SbACP proteins found that the amino acid sequences around the DLS motif were very conserved, and these sequences were the typical feature of the SbACP family, which was the binding site of phosphopantetheine [43]. These results showed a variation in the sequence structure of the SbACPs family, despite a high degree of conservation in key regions. SbACPs might have undergone evolutionary events that resulted in changes in gene structure and function [44, 45].

Genes with similar gene structure and protein structure often have similar functions. In phylogenetic tree analysis, all identified genes in four species were roughly divided into four branches. We found that there was almost always an ACP gene of maize and rice adjacent to a SbACP gene, while the branches of Arabidopsis ACP genes were farther from the SbACPs, suggesting that sorghum was more closely related in evolutionary relationship with maize and rice than Arabidopsis. Generally, the genes under the same branch had high homology and similar functions [46]. We also studied the collinearity of SbACPs in sorghum genome and Arabidopsis, rice and maize genome, respectively. Collinearity analysis showed that there were more homologous ACPs between monocotyledons. These findings suggested that the SbACP genes might function in similar way with ACP genes of maize, which might be the result of the further differentiation of ACP genes on different branches after the differentiation of monocotyledons [47]. *SbACP5* and *SbACP7* shared a large fragment replication, so they were in the same branch of the evolutionary tree, and their gene structure and conserved motif were basically same. The segmented copies of genes tend to have the same function and expression patterns [48]. In this study, different from previous studies, *SbACP5* and *SbACP7* had different response patterns to drought and salt treatment, which might be caused by redundancy of function. Another alternative reason for the different response patterns of the two genes may be related to the differences in their *cis*-acting elements, which endowed them with different functions [49, 50]. Although about 10% of the colinear genes have been found throughout the genome, no tandem duplication existed in SbACPs family, and fragment replication also played only a minor role in the evolution and replication of the SbACPs family, hinting relative independent evolution events of most of SbACPs [49].

In Arabidopsis, ACP genes were divided into two types: plastidial ACP and mitochondrial ACP [35, 42]. In sorghum, ACP genes were also predicted to express in chloroplast and mitochondria. Fluorescence localization verified that all the 5 selected SbACP genes were expressed in chloroplasts, including *SbACP2* and *SbACP4*, which were predicted to localize in chloroplast or mitochondria, indicating the reliability and accuracy of prediction results. But we still could not rule out the possibility of double-localizing for several SbACP genes. On the other hand, Yang et al. systematically analyzed ACP genes of 20 plant species and found that protein sequences (motifs and length) were highly conserved in different ACP branches [42]. Coincidently, Farmer et al. proposed the same opinions and thought that the three-dimensional structure of the acyl carrier proteins was highly conserved, which were folded into a flexible α-helical bundle [42]. In this study, the three-dimensional structure prediction showed the same results with the former researches. Rich α-helix of SbACPs formed α-helical bundle, and further constructed the hydrophobic cavities with structural plasticity, that allowed the accommodation of different lengths of thioester-bound acyl chains [39, 51].

The number and type of *cis*-acting elements in gene promoter region have different regulation effects on regulation of gene expression. Analysis of the 1.5 kb promoter region upstream of SbACPs revealed many TATA-box elements binding to RNA polymerase, which determined the transcription initiation of SbACPs. In addition, the promoter region of SbACPs contained many elements related to ABA, light response and abiotic stress, suggesting that the function of SbACP genes might be diversified. Some previous studies have proven this opinion. For example, seven motifs related to ABA and light-mediated gene regulation in the promoter of ACP (CACTFTPPCA1, DOFCOREZM, GT1CONSENSUS, CAATBOX1, ARR1AT, POLLEN1LELAT52 and GATABOX) in different species were found [42], indicating that ACPs might participate in several biological processes.

In Arabidopsis, the expression level of *AtACP4* (*At4g25050*), a key gene to the synthesis of fatty acids in chloroplast membrane lipids, was positively correlated with the photosynthetic system, which demonstrated that *AtACP4* might play an important role in light, nitrogen, and iron deficiency [14]. *SbACP6*, located in the same cluster as *AtACP4*, had many *cis*-acting elements related to light response in the promoter region, such as AE-box, G-box, GATA-Motif and GT1-motif, which implied that *SbACP6* and *AtACP4* might have similar response patterns. Meanwhile, the identification of MBS, MYB and MYB-like stress-related elements suggested that *SbACP6* may be involved in abiotic stress, which was verified by qRT-PCR [34]. Additionally, previous studies showed that the overexpression of *AtACP5* (At5g27200), a plastid localized protein, could increase the content of saturated fatty acids, reduce the Na^+^/K^+^ ratio, and significantly increase the salt tolerance [14], indicating that fatty acids in plants were associated with abiotic stress. Plastids can be divided into three types: chloroplasts, chromoplasts (or chromatids), and leucoplasts. Most SbACPs proteins displayed obvious expression signals in chloroplasts and they contained many stress-related elements in promoter sequences. Consequently, we inferred that when plants were subjected to abiotic stress, ACP was regulated by *cis*-acting elements (such as MYB, etc.) to increase spatio-temporal expression, and increase the proportion of saturated fatty acids on chloroplast membrane, thus reducing the dissolution of thylakoid grana lamellae [17, 52].

Fatty acid is one of the main components of membrane, and also an important substance for cells to adapt to environmental changes because of the important barrier function of cell membrane [53]. The change of fatty acids in membrane is also a response mechanism to various abiotic stresses [16, 17, 54, 55]. ACPs could react to the external environment stimulation, and further lead to the changes in fatty acid composition and content, implying that ACPs might play a crucial part in response to the adverse environment [16, 17], which was identified by the expression differences of several SbACPs under salt and drought treatments in the current study. Seven of all the nine SbACP genes could response to salt or drought stress with different expression patterns, except *SbACP4* and *SbACP8*. Remarkably, the expression of *SbACP9* was up-regulated from 6 to 24 h after treatment, and showed a rapid and continuous response to drought stress, which suggested that *SbACP9* might function in the drought resistance in a direct way. All these results suggested that ACP genes in sorghum might play an important role in response to various biological process, including light and abiotic stresses, which could provide valuable information for the subsequent functional studies of SbACP genes, and would benefit the breeding of sorghum resistant germplasm.

## Conclusions

In summary, a total of 36 genes encoding SbACP proteins with PP-binding domain were identified in sorghum, maize, rice, and Arabidopsis, which were mainly divided into four families according to the analysis of phylogenetic of ACPs. These ACP family genes were unevenly distributed on six chromosomes. The gene structure and motif composition were also analyzed. The phylogenetic relationship among ZmACP and SbACP proteins were closer, providing a clue to its possible function. It was found that fragment replication might lead to the production of SbACPs genes. Phylogenetic comparison and synteny analysis of ACP genes from 4 typical plant species provided valuable clues about the evolutionary characteristics of ACP gene family members in sorghum. Colinear analysis of ACP in sorghum and other three plants provided information for the evolution of SbACP gene family in sorghum. Besides, these identified SbACP genes were analyzed by *cis*-acting element for a further investigation on their performance in plant growth and stress responses. Gene expression patterns of SbACP genes in leaves under drought and salt stresses were also analyzed. This study of the SbACP family in structure, evolution and expression profiling facilitated the analysis of the SbACP gene function and built a foundation for a better research of the mechanism of abiotic stress tolerance in sorghum.

## Materials and methods

### Identification of ACP gene family in sorghum

The sorghum BTx623 genome dataset, including the CDSs and protein sequences, was downloaded from the Ensembl database (http://plants.ensembl.org/index.html). The hidden Markov model (HMM) was used to query the SbACP protein sequences according to the PP-binding domain (PF00550) in Pfam (http://pfam.xfam.org/) throughout the whole genome using the default parameters in HMMER software. The Hummsearch program in the Linux system was used to search for proteins containing the conserved domain [56], and these sequences were confirmed on SMART, NCBI CDD, and Pfam, respectively. Finally, nine SbACP genes were identified and named according to their position on chromosomes. The ExPASy Proteomics Server software (http://web.expasy.org/protparam/) was used to analyze the protein sequence, protein molecular weight (Mw), and calculate isoelectric point (PI) [57].

### Chromosome localization analysis of ACP family in sorghum

The location of 9 SbACP genes and chromosome length information were extracted through the annotation information of the sorghum genome, and the chromosome location map of genes was drawn by Mapchart software [58].

### Subcellular localization of ACP proteins in sorghum

SbACP protein sequences were submitted to CELLO (http://cello.life.nctu.edu.tw/) to predict the subcellular localization of proteins [59] and then the prediction results were verified by experiments. Specific primers (Table S1) were designed according to the sequence of SbACPs CDS which were used to amplify the whole length of CDS of SbACPs by KOD One TM PCR Master Mix (TOYOBO, Osaka, Japan). The Vector was linearized using *Hind*III and *Bam*HI (NEB, Nebraska, USA). The full length CDSs of 5 selected SbACPs were subcloned into the destination vector with GFP using the ClonExpress® II One Step Cloning Kit (Vazyme, Nanjing, China). The target gene containing the GFP fusion proteins were transferred into Agrobacterium GV3101 and infecting tobacco leaves. After cultured for 48 h of tobacco plants in dark condition at 28℃, the fluorescence images were observed using Leica TCS SP8 (Mannheim, Germany) and A1R HD25 (Nikon, Japan) confocal microscope image system.

### Evolution analysis of ACPs in sorghum

The phylogenetic tree of ACP family proteins in sorghum, maize, and Arabidopsis was constructed by the adjacency joining algorithm in MEGA X software [60] and a bootstrap test (replications) with 1,000 iterations was performed. The obtained evolutionary tree was further modified using the Evolview website (http://www.omicsclass.com/article/671).

### Analysis of ACP family gene structure and motif in sorghum

To analyze the structural information of SbACPs, the annotation information of 9 SbACP genes was extracted. The GSDS2.0 website (http://gsds.gao-lab.org/) were used to analyze the exon and intron structure and draw the gene structures. The MEME (https://meme-suite.org/meme/) was utilized to carry out the motif analysis based on the protein sequences of nine SbACPs.

### Duplication and syntenic analyses of ACPs between sorghum and other species

Multiple Collinearity Scan toolkit (MCScanX) [61] with the default parameters was used to analyze the gene duplication events. To investigate the homology of the ACPs gene family between sorghum and three other species, the Dual Systeny Plotter software (https://github.com/CJ-Chen/TBtools) was used to map the intergenomic collinearity analysis.

### Multi-sequence alignment and three-dimensional structure prediction of sorghum ACP family protein structure

To analyze the conserved domains of the SbACP proteins, clustalw from MEGA X [62] was used for multi-sequence alignment of 9 protein sequences, and then the geneDoc software [63] was used to calculate and analyze the conserved sequences of the SbACP family. To further analyze the protein structure of the SbACP family, the 3D structure of SbACP proteins was predicted according to the protein sequence of SbACPs, and 3D protein models were constructed on the Swiss-Model website (https://swissmodel.expasy.org) by the homologous protein modeling method.

### *Cis*-acting element analysis

To explore the regulation of gene expression, 1.5 kb sequences upstream of the initiation codon of SbACP genes were extracted to analyze the *cis*-acting elements of these genes. Plant CARE (*Cis*-Acting Regulatory Element, http://bioinformatics.psb.ugent.be/webtools/plantcare/html/) was used to further analyze the *cis*-acting elements, and the results were mapped using the GSDS online website (http://gsds.gao-lab.org/).

### Plant materials and treatment

In this study, the sorghum BTx623 was grown in a climate chamber to the two-leaf stage and then transferred to Hoagland nutrient solution. The temperature and light cycle were set at 24 °C with a 14 h/10 h (light and dark) photoperiod. After two days of culture, the seedlings were treated with 400 mM PEG6000 [64] and 150 mM NaCl treatment [65], respectively. The control groups were set for each treatment. The leaves from both the blank control and salt or drought (PEG) treatment with three biological replicates were collected at 0, 6, 12 and 24 h after treatments, then immediately frozen in liquid nitrogen and stored in an ultralow temperature refrigerator at -80 °C for subsequent experiments.

### RNA extraction and qRT-PCR

The RNA was extracted using the ultrapure RNA extraction kit (CWBIO, Taizhou, China), and the HiScript III RT SuperMix for qPCR (+ gDNA Wiper) kit (Vazyme, Nanjing, China) was used for reverse transcription. ChamQ SYBR qPCR Master Mix (LowROX Premixed) kit (Vazyme, Nanjing, China) was used for real-time quantitative PCR analysis. Primer 5.0 was used to design qPCR primers for the ACP gene family, and the sequences of primers were shown in Table S2. The reaction volume was 20µL, and the amplification procedures were: pre-denaturation at 95℃ for 30 s, denaturation at 95℃ for 10 s, annealing at 60℃ for 30 s, and 40 cycles [66]. Each gene was replicated three times biologically and three times technically. *Actin* was used as a control and related gene expression levels were quantified by 2^−ΔΔCt^ [67].

## Supplementary Information

**Additional file 1: Table S1. **The primers for subcellular localization.

**Additional file 2: Table S2. **The primers for qRT-PCR.

## Data Availability

The datasets generated during the current study are available in the Ensemble, https://asia.ensembl.org/index.html and their public access to these databases are open. All data generated or analyzed during this study are included in this article and its supplementary information files.

## Abbreviations

ACPs: Acyl Carrier Protein (s)
qRT-PCR: Quantitative real-time PCR
GSDS: Gene Structure Display Server
MW: Molecular weight
PI: Theoretical isoelectric point
PEG: Polyethylene glycol
Ser: Serine
FAs: Fatty acids
UFAs: Unsaturated FAs
SFAs: Saturated FAs
ER: Endoplasmic reticulum
JGI: The United States Department of Energy joint Genome Research Institute
DSL: Asp-Ser-Leu
HMM: Hidden Markov model
PP-binding: Phosphopantetheine, or pantetheine 4'phosphate-binding
MCScanX: Multiple Collinearity Scan toolkit
